# Influences of defective interphase and contact region among nanosheets on the electrical conductivity of polymer graphene nanocomposites

**DOI:** 10.1038/s41598-024-63981-1

**Published:** 2024-06-08

**Authors:** Yasser Zare, Muhammad Tajammal Munir, Kyong Yop Rhee

**Affiliations:** 1 Biomaterials and Tissue Engineering Research Group, Department of Interdisciplinary Technologies, Motamed Cancer Institute, Breast Cancer Research Center, ACECR, Tehran, Iran; 2 College of Engineering and Technology, American University of the Middle East, 54200 Egaila, Kuwait; 3 Department of Mechanical Engineering (BK21 four), College of Engineering, Kyung Hee University, Yongin, Republic of Korea

**Keywords:** Polymer graphene nanocomposites, Imperfect interfacial adhesion, Conductivity, Contact region, Tunneling mechanism, Engineering, Materials science

## Abstract

In the current article, a defective interface is characterized by “D_c_,” representing the smallest diameter of nanosheets crucial for effective conduction transfer from the conductive filler to the medium, and by “ψ” as interfacial conduction. These parameters define the effective aspect ratio and operational volume fraction of graphene in the samples. The resistances of the graphene and polymer layer in contact zones are also considered to determine the contact resistance between adjacent nanosheets. Subsequently, a model for the tunneling conductivity of composites is proposed based on these concepts. This innovative model is validated by experimental data. Additionally, the effects of various factors on the conductivity of the composites and contact resistance are analyzed. Certain parameters such as filler concentration, graphene conductivity, interfacial conduction, and “D_c_” do not affect the contact resistance due to the superconductivity of the nanosheets. However, factors like thin and large nanosheets, short tunneling distance (d), high interfacial conduction (ψ), low “D_c_,” and low tunnel resistivity (ρ) contribute to increased conductivity in nanocomposites. The maximum conductivity of 0.09 is obtained at d = 2 nm and ψ = 900 S/m, but d > 6 nm and ψ < 200 S/m produce an insulated sample. Additionally, the highest conductivity of 0.11 S/m is achieved with D_c_ = 100 nm and ρ = 100 Ω m, whereas the conductivity approaches 0 at D_c_ = 500 nm and ρ = 600 Ω m.

## Introduction

Electrical conductivity in nanocomposites, hereafter referred to as conductivity, is achieved by incorporating conductive nanofillers, including carbon blacks, carbon nanotubes (CNT), and graphene, into polymer matrices. Among these nanoparticles, graphene stands out due to its superior mechanical rigidity, high thermal conductivity, and outstanding electrical conductivity^1–11^. Consequently, graphene nanosheets offer significant advantages in enhancing the electrical properties of polymer matrices. Initial studies in this domain have concentrated on producing graphene-based materials with minimal percolation threshold^12–16^. Typically, the percolation threshold is associated with the filler aspect ratio in the nanocomposites, which depends on the degree of dispersion and the aggregation/agglomeration of nanoparticles within the polymer matrix^17–22^. Thus, the extent of graphene dispersion, to form continuous networks within a polymer matrix, is crucial for improving conductivity. It was indicated that agglomeration of nanofillers into the polymer matrix can reduce the thermo-mechanical properties and piezoelectric sensitivity of nanocomposite^23,24^. So, it is attempted to provide a uniform dispersion of nanoparticles in the polymer nanocomposites to achieve the significant performances.

New research on polymer-graphene nanocomposites can explore conductivity from various perspectives. For instance, electron transfer through the contact zones between adjacent nanosheets relies on quantum mechanics^25–29^. Therefore, the conductivity does not necessitate the physical connection of nanosheets within the networks, as electrons can move through small tunneling regions. Consequently, the onset of percolation in graphene and its conductivity are determined by the tunneling effect, though this critical factor has often been overlooked in studies. Indeed, the consideration of contact zones can justify the low percolation threshold and high conductivity observed.

Nanocomposites often suffer from inadequate adhesion at the polymer-filler interface, attributed to poor compatibility between the medium and nanofillers^30,31^. Such subpar interfacial properties compromise the nanocomposite performance, including stiffness, as the advantageous characteristics of nanofillers cannot be fully transferred to the matrices through a weak interface^32,33^. Considering the interfacial properties, previous works have proposed the minimum length of platelets necessary for completely transferring stress from the medium to the clay (L_c_) and have defined interfacial shear strength (τ), as well as the effective aspect ratio and effective volume fraction^34^. The strength of the interface can also influence the percolation threshold and conductivity, since the conductivity must be transferred from the super-conductive filler to the medium. In essence, a robust interface can efficiently distribute the filler conductivity to the matrices, whereas a poor interface fails to facilitate this transfer. However, the specific focus on these aspects in graphene nanocomposites has been scant.

In this paper, the concept of an incomplete interface is represented by “D_c_,” denoting the minimum diameter of nanosheets essential for effective conduction transfer from the conductive filler to the medium, and by “ψ” as interfacial conduction. These parameters are used to define the effective aspect ratio and effective volume fraction of graphene. Additionally, the resistances of the graphene portion and the polymer layer in contact zones are defined to determine the contact resistance between adjacent nanosheets. A model for the conductivity of graphene samples is developed based on these parameters. This established model is validated against experimental data, and the effects of various factors on the conductivity and contact resistance are assessed.

Although some models were developed for the conductivity of polymer graphene systems by interphase and tunneling effects, the defective interface was ignored in the previous articles^35–38^. Actually, the previous articles have considered the perfect interphase in the nanocomposites, whereas most nanocomposites include the imperfect interphase. The current article presents a novel approach to enhance the understanding of electrical conductivity in polymer-graphene nanocomposites, focusing on the concept of defective interface. This innovative framework characterizes interfaces using parameters like the minimum effective diameter of nanosheets (D_c_) and interfacial conduction (ψ), diverging from traditional models by emphasizing the impact of incomplete adhesion at the polymer-filler interface on conductivity. A significant advancement is the comprehensive model developed for predicting tunneling conductivity, incorporating quantum mechanics to account for electron transfer through contact zones without requiring physical connection between nanosheets. The predictive capability of model is validated by experimental data, with a detailed parametric analysis revealing how various factors, including graphene concentration and conductivity, influence conductivity and contact resistance. This exploration into the role of interfacial adhesion and contact zones in determining conductivity opens new avenues for optimizing nanocomposite materials, marking a considerable contribution to material science by blending theoretical innovation with empirical validation.

## Modeling views

A poor interface cannot withstand high shear stress, leading to yielding or debonding during the loading process. Thus, interfacial shear stress indicates inadequate development of normal stress, requiring a large area for normal stress to effectively utilize the filler potential^34^. In this condition, a broad range of filler lengths is insufficiently stressed due to defective interfacial adhesion, compromising the reinforcing efficiency of nanoparticles.

Similarly, the impact of deficient interfacial adhesion on conductivity can be elucidated using the same principle. “D_c_” is the minimum diameter of nanosheets necessary to transfer the complete conduction from graphene nanosheets to the insulating polymer. Actually, “D_c_” determines the level of conductivity that can be transferred to the medium in the case of imperfect interface/interphase.

“D_c_” is expressed as:

1 $$ D_{c} = \frac{{\sigma_{{f_{} }} t}}{2\psi } = \frac{{\sigma_{{f_{} }} \alpha_{} D}}{2\psi } $$

where “σ_f_”, “D”, and “t” represent the conductivity, diameter, and thickness of nanosheets, respectively, “α” is defined as the inverse aspect ratio (α = t/D), and “ψ” denotes interfacial conduction.

**Figure 1:**
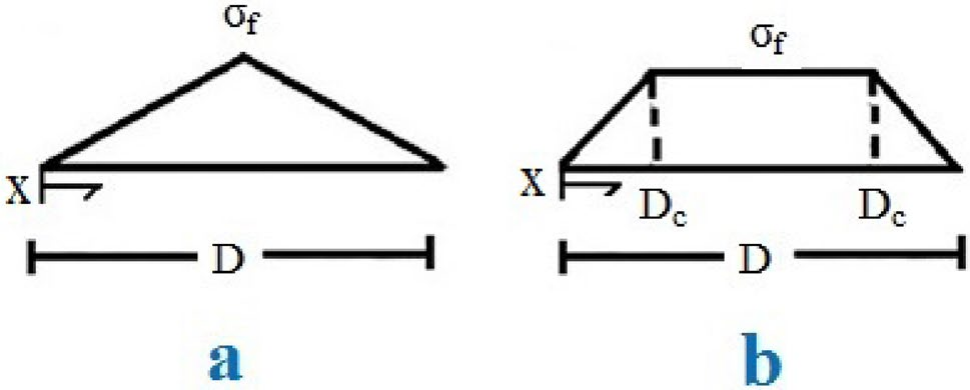
Normal conductivity at two states: (**a**) x < 2D_c_ and (**b**) x > 2D_c_.

Figure 1 depicts sketches of normal conductivity (σ) in a nanosheet at two different positions. In the first case ($x \le D_{c}$), the optimal diameter of nanosheets does not achieve “σ_f_”; however, in the second scenario ($D_{c} < x$), “σ” can reach “σ_f_”, representing the maximum conductivity of nanoparticles that effectively transfer the filler conductivity to the medium.

The average normal conductivity ($\overline{\sigma }$) equals “σ_f_” when graphene nanosheets are perfectly attached to the medium. Nevertheless, “($\overline{\sigma }$)” is lower than “σ_f_” when assuming partial interfacial attachment, which reduces the effective diameter of nanosheets ($D_{eff}$), as detailed in Eq. (2).

2 $$ \overline{\sigma } D = \sigma_{f} D_{eff} $$

The effective inverse aspect ratio ($\alpha_{eff}$) and effective volume fraction ($\phi_{eff}$) of nanosheets are reduced by weak interfacial bonding. The terms $\alpha_{eff}$ and $\phi_{eff}$, assuming $x < 2D_{c}$ and $x > 2D_{c}$ regions as illustrated in Fig. 1, and reported in^34^, also described in Eqs. (3) and (4), respectively.

3 $$ \alpha_{eff} = \alpha \left( {\frac{{8D_{c}^{2} }}{{D^{2} }} + 1} \right) $$

4 $$ \phi_{eff} = \phi_{f} \left[ {\frac{1}{2} + \left( {\frac{{D - 2D_{c} }}{{D^{2} }}} \right)(D - D_{c} )} \right] $$

where “$\phi_{f}$” is filler volume portion.

Exchanging of “D_c_” from Eq. (1) into Eqs. (3) and (4) suggests:

5 $$ \alpha_{eff} = \alpha \left( {\frac{{2\sigma_{f}^{2} \alpha^{2} }}{{\psi^{2} }} + 1} \right) $$

6 $$ \phi_{eff} = \phi_{f} \left[ {\frac{1}{2} + \left( {1 - \frac{{\sigma_{{f_{} }} \alpha_{} }}{\psi }} \right)\left( {1 - \frac{{\sigma_{{f_{} }} \alpha_{} }}{2\psi }} \right)} \right] $$

which undoubtedly handle the percolation inception and conductivity.

It was mentioned that electrons can be transferred through the contact zones between adjacent sheets. The contact resistance is defined by the resistances of the nanosheets and the insulating polymer layer in these areas. Accordingly, the contact resistance comprises the resistances of graphene portion (R_g_) and polymer layer (R_t_) in the contact zones as:

7 $$ R_{c} = R_{g} + R_{t} $$

“R_g_” and “R_t_”^39^ were defined as:

8 $$ R_{g} = \frac{1}{{2t\phi_{f} \sigma_{f} }} $$

9 $$ R_{t} = \frac{\rho d}{{tD}} $$

where “ρ” is tunnel resistivity and “d” is tunneling distance/length.

The contact resistance is suggested by combination of these equations as:

10 $$ R_{c} = \frac{1}{{2t\phi_{f} \sigma_{f} }} + \frac{\rho d}{{tD}} $$

Assuming the operative filler portion due to incomplete interfacial adhesion (Eq. 6) develops the latter equation to:

11 $$ R_{c} = \frac{1}{{2t\phi_{f} \left[ {\frac{1}{2} + \left( {1 - \frac{{\sigma_{{f_{} }} \alpha_{} }}{\psi }} \right)\left( {1 - \frac{{\sigma_{{f_{} }} \alpha_{} }}{2\psi }} \right)} \right]\sigma_{f} }} + \frac{\rho d}{{tD}} $$

which expresses the contact resistance by many parameters attributed to graphene, contact zones and interfacial zones.

A simple model can define the contact resistance influence on the conduction. The conductivity of CNT-containing samples was proposed^40^ as:

12 $$ \sigma = \frac{{lw_{f}^{2x + 1} }}{{2\pi r^{2} (R_{f} + R_{c} )}} $$

where “w_f_”, “r”, “l” and “R_f_” show the weight fraction, radius, length, and inherent resistance of CNT, in that order and “x” is a parameter. The experimented levels of conductivity approved the predictability of this equation^40^.

This model can be progressed to estimate the tunneling conductivity of graphene products assuming operative filler portion and graphene dimensions as:

13 $$ \sigma = \frac{{D\phi_{eff}^{2x + 1} }}{{2\pi t^{2} (R_{f} + R_{c} )}} $$

where “R_f_” is graphene inherent resistance calculated by:

14 $$ {\text{R}}_{{\text{f}}} = \frac{1}{{t\sigma_{f} }} $$

Substituting of “$\phi_{eff}^{}$” from Eq. (6), “R_f_” from Eq. (14) and “R_c_” from Eq. (11) into Eq. (13) suggests a developed model, which appearances the stimuli of graphene properties, extent of interfacial adhesion, tunneling size and contact resistance on the conductivity.

## Results and discussion

### Evaluation of developed model by experimental data

**Figure 2:**
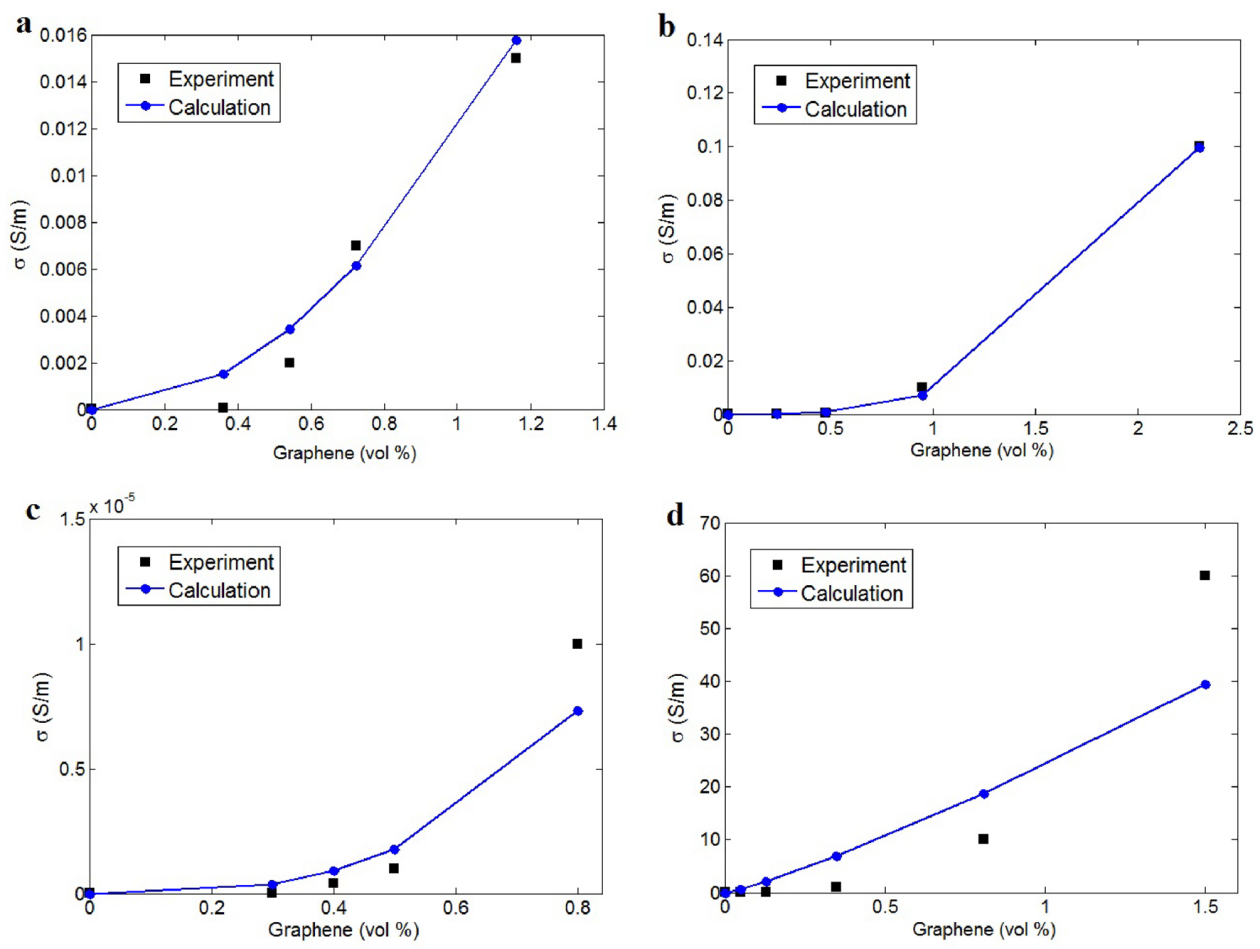
Experimental results and predictions by Eq. (13) for (**a**) PVA^41^, (**b**) ABS^42^, (**c**) PVDF^14^ and (**d**) PS^13^ graphene samples.

The developed approach proves to be practical for estimating the conductivity and principal parameters in certain samples. Table 1 displays the samples along with their characteristics. By applying conductivity measurements to the novel model (Eq. 13), it is possible to deduce these parameters. The conductivity predictions for the reported samples are illustrated in Fig. 2a–d, clearly demonstrating that the current model can accurately calculate conductivity for various examples. Therefore, the new model is capable of estimating the conductivity of composites through interfacial bonding and contact zones, with the calculations of different parameters by the developed equations also presented in Table 1. The developed model nonlinearly correlates the conductivity to graphene concentration. However, it seems that the variation of conductivity with graphene amount is almost linear in Fig. 2d for PS/graphene sample, because it was plotted at low graphene concentrations. Also, it has the lowest levels of “ρ”, “R_c_” and “x” (as shown in Table 1) increasing the conductivity at very low graphene concentrations.

**Table 1:** The detailed data for examples and the calculations of factors by the equations.

Samples [Ref.]	t (nm)	D (μm)	d (nm)	ψ (S/m)	D_c_ (nm)	ρ (ohm.m)	R_f_ (ohm)	R_c_ (ohm)	x
PVA/graphene^41^	2	2	9	500	200	450	5*10^3^	1.00*10^9^	0.5
ABS/graphene^42^	1	4	3	500	100	300	10^4^	2.25*10^8^	1.0
PVDF/graphene^14^	1	2	3	300	167	3*10^4^	10^4^	4.50*10^10^	1.0
PS/graphene^13^	1	4	8	50	100	80	10^4^	1.60*10^8^	0.1

The largest tunnels and the highest “D_c_” values are observed in the PVA/graphene sample. Furthermore, the highest levels of tunnel resistivity and “R_c_” are found in the PVDF/graphene sample, which exhibits the lowest conductivity among the examples (Fig. 2c). Conversely, the lowest levels of tunnel resistivity and contact resistance are seen in the PS/graphene nanocomposite, indicating the highest conductivity among the reported samples (Fig. 2d). Thus, conductivity is significantly related to contact resistance, as it governs the transfer of electrons between adjacent nanosheets. Additionally, the “x” value varies from 0.5 to 1 for the reported samples. Values of 2x + 1 ranged from 2.7 to 5.3 for polymer CNT nanocomposites^40^, indicating that the “x” levels in graphene nanocomposites are lower than those in CNT nanocomposites.

### Analysis of parameters

**Figure 3:**
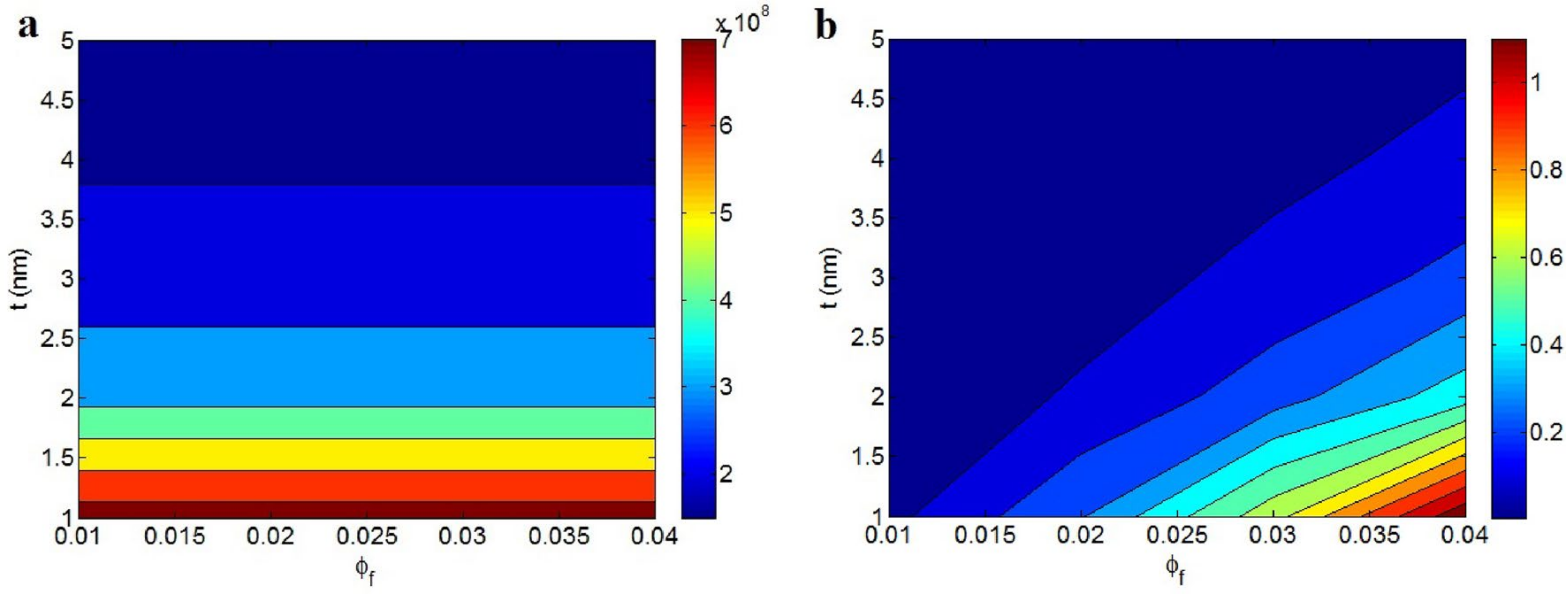
(**a**) “R_c_” and (**b**) conductivity by “$\phi_{f}$” and “t” at σ_f_ = 10^5^ S/m, D = 2 μm, d = 5 nm, ψ = 400 S/m, ρ = 300 Ω m and x = 0.5.

Figure 3a presents the effects of “$\phi_{f}$” and “t” on the contact resistance, as outlined in Eq. (11), through a contour plot with average values of d = 5 nm, D = 2 μm, σ_f_ = 10^5^ S/m, ψ = 400 S/m and ρ = 300 Ω m. The contact resistance does not depend on “$\phi_{f}$”, but exhibits an inverse relationship with “t”. Thus, thin nanosheets demonstrate high contact resistance, while thick nanosheets exhibit lower one. This is because thin nanosheets decrease the contact area in the contact region, thereby increasing contact resistance. However, the volume fraction of nanosheets does not significantly influence resistance, as graphene considerable conductivity negates any potential resistance within the contact zones.

Figure 3b also illustrates the effects of “${\phi }_{f}$” and “t” on conductivity, as outlined in Eq. (13) (x = 0.5). The highest conductivity, approximately 1.1 S/m, is observed at ${\phi }_{f}=0.04$ and t = 1 nm. However, low “${\phi }_{f}$” and high “t” decrease conductivity. Thus, a large quantity of thin nanosheets can enhance conductivity, but a small quantity of thick graphene does not have the same effect. The dependency of conductivity on filler concentration is evident since it dictates the concentration of the conductive phase in the nanocomposites. Nonetheless, even though thin nanosheets produce high contact resistance, they result in the highest conductivity. This suggests that thin nanosheets reduce “D_c_,” which positively influences the effective inverse aspect ratio and filler volume fraction. Consequently, thin nanosheets are crucial for the transfer of conduction from nanoparticles to the polymer medium. Therefore, it is advisable to use thinner nanosheets to achieve higher conductivity. Previous studies^13,42^ have also demonstrated the beneficial effects of thin nanosheets on percolation threshold and conductivity.

**Figure 4:**
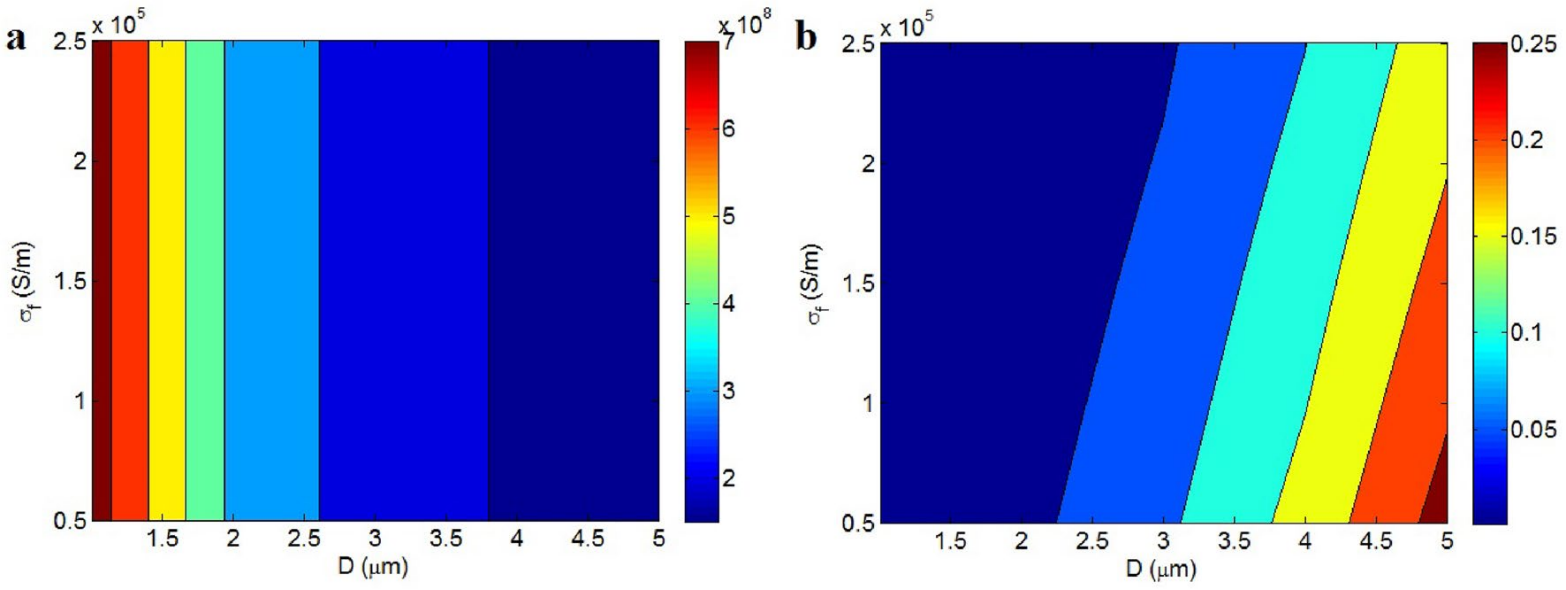
Effects of “D” and “σ_f_” on (**a**) “R_c_” and (**b**) conductivity ($\phi_{f}$ = 0.01, d = 5 nm, t = 2 nm, ψ = 400 S/m, ρ = 300 Ω m and x = 0.5).

Figure 4a also showcases the effects of “D” and “σ_f_” on the contact resistance at t = 2 nm, $\phi_{f}$ = 0.01, d = 5 nm, ψ = 400 S/m and ρ = 300 Ω m. A lower "D" leads to increased contact resistance, while “σ_f_” does not have an impact. Specifically, smaller nanosheets result in higher contact resistance, although the conductivity of graphene itself does not control the contact resistance. Larger nanosheets expand the contact area between adjacent nanosheets, thus reducing resistance, because contact resistance inversely correlates with the contact area due to graphene nanosheets spanning the contact zones. However, the substantial conductivity of graphene nanosheets does not influence resistance, as the resistance within the contact zones is significantly lower than that of the surrounding polymer layer. Essentially, the resistance offered by conductive graphene is minimal and does not alter the contact resistance. Therefore, the equation accurately reflects the effects of “D” and “σ_f_” on the contact resistance “R_c_”.

The conductivity at dissimilar ranks of “D” and “σ_f_” is also shown in Fig. 4b (x = 0.5). The highest conductivity of 0.25 S/m is achieved by D = 5 μm and σ_f_ = 0.5*10^5^ S/m, although an insulated product is perceived at D < 2.25 μm. Consequently, large nanosheets and low graphene conductivity lead to desirable conductivity. In contrast, small nanosheets do not enhance conductivity. Large nanosheets reduce the “D_c_,” indicating effective interfacial properties. A lower “D_c_” increases the effective filler fraction and reduces the effective inverse aspect ratio. Moreover, as noted, large nanosheets can result in lower contact resistance within the nanocomposites. For these reasons, it is logical to recognize a direct relationship between conductivity and the diameter of graphene nanosheets.

Graphene conductivity adversely impacts the conductivity in scenario of partial interfacial adhesion, as a high level of filler conductivity cannot be effectively transferred to the medium under such conditions. Indeed, the significant disparity between the conductivity of the polymer and that of graphene necessitates perfect interfacial adhesion to benefit conductivity. Elevated filler conductivity leads to an increase in “D_c_,” which detrimentally influences the effective inverse aspect ratio and effective filler fraction. Consequently, it is anticipated that high filler conductivity results in reduced conductivity.

**Figure 5:**
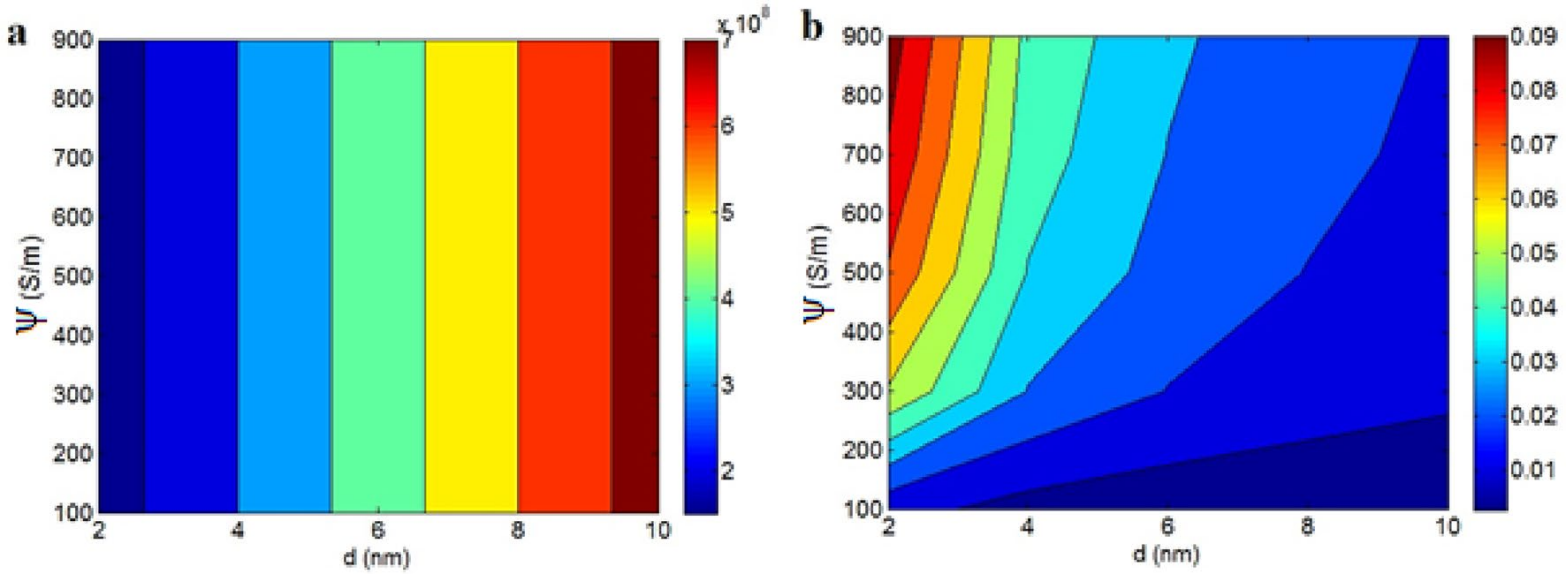
The estimations of (**a**) “R_c_” and (**b**) conductivity at various amounts of “d” and “ψ”.

The dependency of contact resistance on “d” and “ψ” is also exhibited in Fig. 5a. The contact resistance directly relates to “d”, but “ψ” cannot change it. A high contact resistance of 7*10^8^ Ω is estimated at d = 10 nm, but “R_c_” value of 1.5*10^8^ Ω is observed at d = 2 nm. Thus, the tunneling distance directly influences the contact resistance, while interfacial conduction does not control it. The contact zones consist of a polymer film sandwiched between two graphene nanosheets. However, it's the resistance of the polymer film that predominantly dictates the contact resistance due to the superconductivity of the graphene nanosheets. A larger tunnel indicates a thicker polymer layer in the contact zones, substantially increasing the contact resistance. Conversely, interfacial conduction only impacts the effective filler concentration via “D_c_” (Eq. 1). Nevertheless, the high conductivity of the nanosheets diminishes its effect on the contact resistance (Rg in Eq. 8), as the graphene nanosheets do not significantly impede electron flow. Therefore, interfacial conduction does not alter the contact resistance, as demonstrated by the provided illustration.

Figure 5b represents the discrepancies of conductivity at unalike heights of “d” and “ψ”. The uppermost conductivity of 0.09 is detected at d = 2 nm and ψ = 900 S/m, though an insulated sample is shown at d > 6 nm and ψ < 200 S/m. Accordingly, the shortest tunneling distance and the highest interfacial conduction yield the most favorable conductivity. A smaller tunneling distance decreases the contact resistance, leading to the expectation that more electrons are transferred through tunnels, thereby enhancing conductivity. However, a larger tunnel encompasses dense insulating layers in the contact zones, significantly impeding electron movement and reducing conductivity. This correlation between a nanocomposite conductivity and tunneling size has been observed in earlier studies^27,43^. Furthermore, high interfacial conduction suggests strong polymer-filler bonding, facilitating the efficient transfer of filler conduction to the polymer medium and thus improving conductivity. Indeed, a high “ψ” positively influences “D_c_”, the effective inverse aspect ratio, and the effective filler fraction, contributing to increased conductivity in nanocomposites.

**Figure 6:**
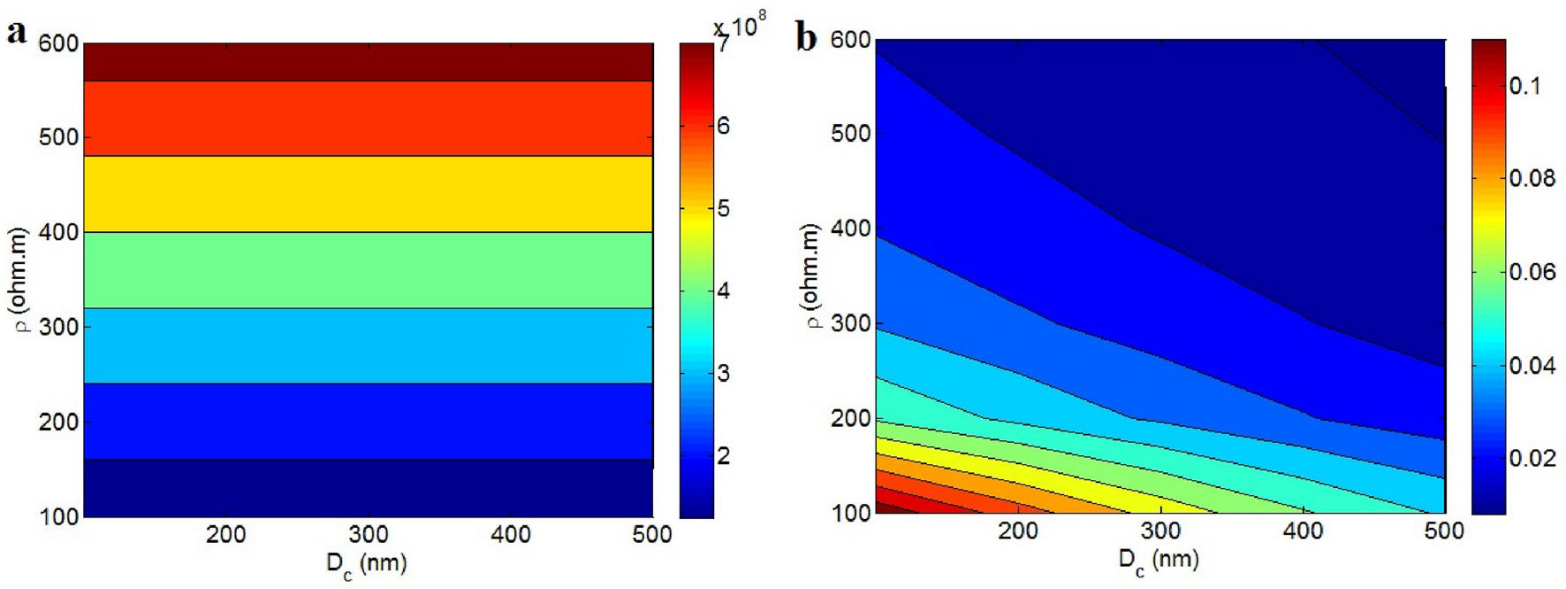
(**a**) “R_c_” and (**b**) conductivity at unlike ranks of “D_c_” and “ρ”.

The inspirations of “D_c_” and “ρ” on the contact resistance are displayed in Fig. 6a. The resistance directly links on “ρ”, but “D_c_” cannot affect it. The highest value of contact resistance is observed at the highest level of “ρ”, indicating that tunnel resistivity significantly influences contact resistance. “D_c_” can alter the effective filler concentration, and contact resistance depends on the operative filler fraction. However, the high conductivity of the filler negates its effect on the “R_g_” term (Eq. 8). Essentially, the substantial conductivity of graphene nanosheets mitigates the influence of various parameters associated with graphene nanosheets on contact resistance, as they do not contribute to high resistance in the contact zones. On the other hand, the polymer tunnel resistivity introduces significant resistance, effectively governing the contact resistance. Hence, the developed equation for contact resistance accurately reflects the effects of “D_c_” and “ρ” on contact opposition.

Conductivity as a function of “D_c_” and “ρ” is depicted in Fig. 6b. “D_c_” and “ρ” have inverse effects on conductivity. The highest conductivity, 0.11 S/m, is achieved with D_c_ = 100 nm and ρ = 100 Ω m, whereas conductivity approaches 0 at D_c_ = 500 nm and ρ = 600 Ω m. Therefore, low levels of “D_c_” and tunnel resistivity are conducive to desirable conductivity.

A small “D_c_” indicates strong interfacial linkage, leading to efficient conduction transfer from graphene to the polymer medium. Consequently, a lower “D_c_” reduces the effective inverse aspect ratio and increases the effective filler amount, thereby enhancing conductivity. On the contrary, high tunnel resistivity limits electron transport in the contact zones, meaning strong tunnel resistivity does not significantly facilitate electron movement within filler networks, thus diminishing conductivity. In summary, the established model appropriately highlights the positive influences of “D_c_” and “ρ” on conductivity.

**Figure 7:**
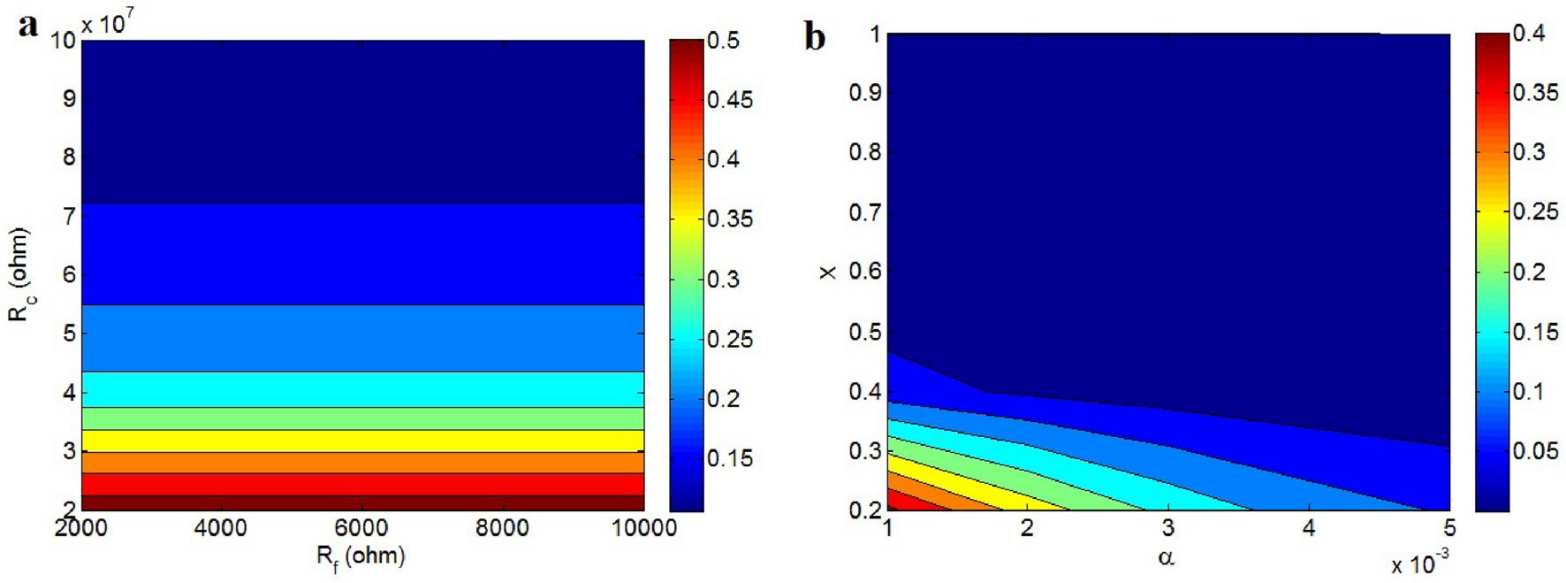
Dependencies of conductivity on (**a**) “R_f_” and “R_c_” and (**b**) “α” and “x” based on Eq. (13).

Figure 7a illustrates the impact of intrinsic graphene resistance and contact resistance on conductivity. It demonstrates that graphene resistance does not alter conductivity, whereas conductivity largely relies on contact resistance. The primary reason for this occurrence is that contact resistance exceeds graphene resistance. Essentially, the minimal level of graphene resistance is insufficient to influence conductivity. Actually, “R_f_” varies at 2000–10,000 Ω, while “R_c_” changes at 2–10*10^7^ Ω. It is clear that “R_f_” is much smaller than “R_c_” due to the high conductivity of graphene nanosheets. So, “R_f_” cannot change the conductivity. However, significant contact resistance, primarily resulting from the insulating polymer medium, substantially impedes conduction.

Figure 7b depicts the conductivity at various levels of “α” and “x”. The highest conductivity, 0.4 S/m, is achieved at α = 0.001 and x = 0.2, whereas values of x > 0.5 markedly reduce conductivity. Therefore, optimal conductivity is reached at the lowest values of the inverse aspect ratio and “x”, with higher “x” values leading to an insulating nanocomposite.

A low inverse aspect ratio (α) has been associated with reduced percolation levels in nanocomposites, as indicated in prior studies^44^. Furthermore, a small α leads to a reduced “D_c_,” which in turn results in a higher effective filler concentration due to the increased interfacial area. Consequently, a lower α effectively enhances conductivity by fostering desirable interfacial properties. On the other hand, a higher “x” adversely impacts conductivity by diminishing the influence of effective filler concentration on conductivity (Eq. 13). Conversely, a lower “x” value amplifies the contribution of filler concentration, thereby improving conductivity. Based on these insights, the model adequately demonstrates the influence of α and “x” on conductivity.

## Conclusions

The challenges of incomplete interfacial adhesion and contact resistance between adjacent nanosheets were acknowledged, leading to the development of a comprehensive model for conductivity. This model, underpinned by established equations, has been validated through experimental data and parametric analysis. It effectively predicts conductivity by considering factors such as interfacial adhesion and contact zones. The calculations derived from the developed equations are in strong agreement with empirical observations. Certain parameters, including filler concentration, graphene conductivity, interfacial conduction, and “D_c_,” do not significantly affect the contact resistance “R_c_”, due to the superior conductivity of nanosheets, which minimizes contact resistance. Conversely, configurations involving thin and small nanosheets, extensive tunnels, and high tunnel resistivity result in increased contact resistance within nanocomposites. Furthermore, configurations with thin and large nanosheets, elevated filler concentration, lower graphene conductivity, reduced tunneling distance, enhanced interfacial conduction, diminished “D_c_,” and decreased tunnel resistivity promote desirable conductivity. Overall, the negligible intrinsic resistance of graphene does not directly modify conductivity; instead, the predominant factor influencing conductivity is the high contact resistance. The influences of various factors on both contact resistance and conductivity have been thoroughly analyzed to validate the accuracy of the proposed equations and model.

## Data Availability

The data that support the findings of this study are available on request from corresponding author.
